# Assessing Intestinal Health. *In Vitro* and *Ex vivo* Gut Barrier Models of Farm Animals: Benefits and Limitations

**DOI:** 10.3389/fvets.2021.723387

**Published:** 2021-11-23

**Authors:** Federico Ghiselli, Barbara Rossi, Andrea Piva, Ester Grilli

**Affiliations:** ^1^Servizio Produzioni Animali e Sicurezza Alimentare, Dipartimento di Scienze Mediche Veterinarie, University of Bologna, Bologna, Italy; ^2^Vetagro S.p.A., Reggio Emilia, Italy; ^3^Vetagro Inc., Chicago, IL, United States

**Keywords:** intestinal health, gut barrier, farm animal, *ex vivo* model, *in vitro* model

## Abstract

Animal performance is determined by the functionality and health of the gastrointestinal tract (GIT). Complex mechanisms and interactions are involved in the regulation of GIT functionality and health. The understanding of these relationships could be crucial for developing strategies to improve animal production yields. The concept of “gut health" is not well defined, but this concept has begun to play a very important role in the field of animal science. However, a clear definition of GIT health and the means by which to measure it are lacking. *In vitro* and *ex vivo* models can facilitate these studies, creating well-controlled and repeatable conditions to understand how to improve animal gut health. Over the years, several models have been developed and used to study the beneficial or pathogenic relationships between the GIT and the external environment. This review aims to describe the most commonly used animals' *in vitro* or *ex vivo* models and techniques that are useful for better understanding the intestinal health of production animals, elucidating their benefits and limitations.

## Introduction

Intestinal health is a complex concept, and several biological and mechanical factors and structures interact to affect intestinal health (1). The gut barrier is one of the main components, but there is no assay or model to completely and accurately recreate the thousands of interactions that occur in the gastrointestinal tract (GIT). *In vitro* and *ex vivo* models can be a simplified and controlled way to clarify specific interactions that involve the GIT barrier. There is a direct relationship between animal performance and a “healthy” gastrointestinal tract, but there is no clear definition of “gut health” (2). Proper nutrient digestion and absorption, a stable microbiome, good mucus layer development, and barrier function, and mucosal immune responses are the main functions of a healthy intestine. In particular, the gut epithelium is constantly exposed to foreign antigens and microorganisms. A healthy intestinal barrier allows the maintenance of mucosal immune homeostasis and prevents the onset of uncontrolled inflammation, which can lead, in the worst-case scenario, to death (3). The GIT barrier also plays a crucial role in maintaining a homeostatic relationship with immune cells and the microbiota. Intestinal epithelial cells are influenced by the microbial environment and can produce molecules, such as cytokines or chemokines, antimicrobial peptides, and hormones (4, 5). The gut microbiota also interacts with the host immune system largely through the gut-associated lymphoid tissue system (6, 7), and in many ways, the immune system can distinguish good bacteria from foes. The enteric nervous system (ENS) is also important for maintaining homeostasis and it is involved in the host-microbiome response. It helps with peristalsis in the gut, hormone secretion, neurotransmitter release, and signaling to the central nervous system (8).

The intestine is a very complex organ in which different components achieve distinct physiological functions in a highly integrated and regulated fashion. Relationships involve a complex network of hormones and cross-talk between cells in all the compartments. *In vitro* and *ex vivo* models are required to get a full description and understanding of the mechanisms and relationships between the various components. Understanding the mechanisms and interactions that affect intestinal health would be the key to find new strategies to improve animal production yields and welfare. Wu et al. (9), for example, provided an extensive review about the endogenous host defense peptides and they analyzed how them can influence the intestinal health of animals. Moreover, they also analyzed how these peptides can be used as antibiotic alternatives. *In vitro* or *ex vivo* models are also needed to reduce the ethical issues and expenses associated with the use of animals in experiments (10). In this context the 3Rs approach (11) is very useful for aiming at “Replace” animals used in experiments with non-sentient alternatives; “Reduce” the number of animals employed; and “Refine” animal experiments to cause minimum distress and pain. An ideal animal model to study gut health should contain all the gut epithelial cell types of the animal considered in the study, and these cells should have the ability to be cultured for the defined assay time without losing their *in vivo* characteristics. Then, the model should also represent the biochemical environment that comprises the epithelium/immune system or ENS crosstalk. *In vitro* models can create well-controlled and repeatable conditions with some limitations. Instead, *ex vivo* models refer to experiments conducted in or on the tissue directly derived from animals (12) but with a shorter lifespan and less reproducibility.

For human models, significant work has been done. Costa et al. (13) have well summarized the current methodologies used to develop human intestinal *in vitro* models and analyzed the future perspective. Are these models available for farm animals? It is possible to recreate the animal counterpart?

As for as farm animals, a few models and cell lines are available. Different techniques and intestinal models to mimic the animal *in vivo* conditions have been developed over the years (12), but the development of more complex systems could be the key to better understand animal intestinal physiology and find ways to improve animal welfare, intestinal health, and production yields. This review aimed to analyze and clarify the benefits and limitations of the main *in vitro* or *ex vivo* models available for farm animals and the techniques useful to understand the relationships between animal intestinal health and gut barrier, clarifying their benefits and limitations.

## *In vitro* Cell-Based Models

**Figure 1:**
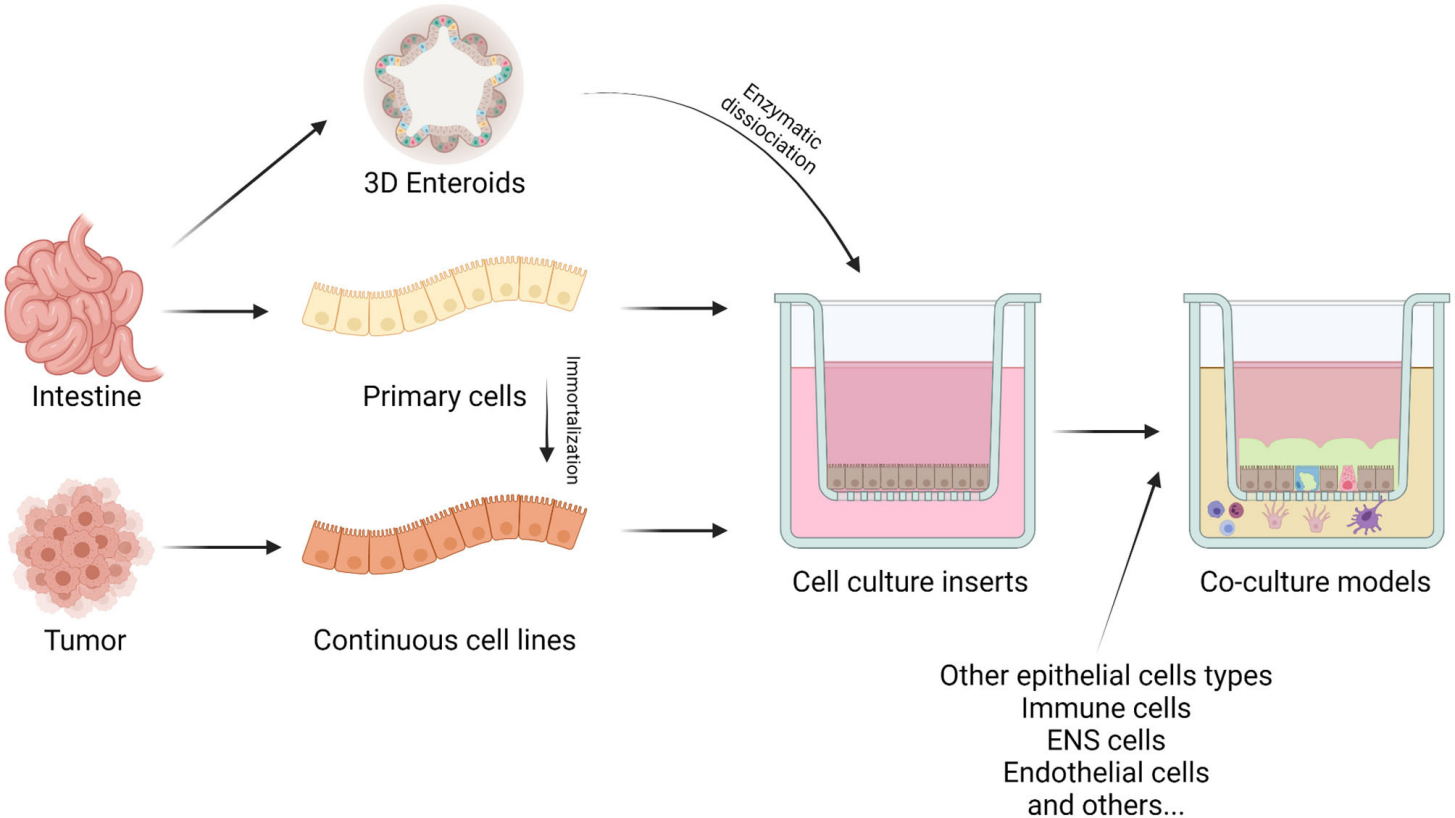
The figure represents the different cell sources for *in vitro* studies. Epithelial cells can derive directly from intestinal tissue or from tumors. The intestine allows the isolations of primary cells or stem cells to produce enteroids and organoids. On the other hand, intestinal tumors generate continuous cell cultures. These can also be obtained by immortalization techniques of primary cells. Cells can be seeded on specific inserts, alone or in co-culture with other cell types to create either monolayers or a more representative intestinal barrier model, respectively. Created with BioRender.com.

To study animal gut barrier health, it is important to have a standardized method that can assess the functionality of this barrier (14). Permeable membranes are the most common supports used to replicate the intestinal barrier configuration (15). This system grants access to both the apical and basolateral compartments. Cells create the intestinal barrier, growing as a monolayer on a permeable membrane (i.e., Transwell® filters), and with this approach, it is possible to mimic the *in vivo* intestinal barrier physiology and functionality (16). In Figure 1 the different ways to create an *in vitro* model are summarized.

Caco-2 cells are the “gold standard” model for *in vitro* intestinal barrier assays, even for animals (17). These cells are derived from a human colorectal carcinoma (18), and at the early stage of culture, they are undifferentiated. When confluent, they form a polarized monolayer joined by tight junctions (TJs), and they express apical microvilli (17, 19, 20). Differentiated Caco-2 cells have most of the morphological and functional characteristics of absorptive intestinal cells, despite their colonic origin (21). These cells do not produce mucus, and they create a replicable population among studies (22). The relatively wide variation of different culture conditions, protocols, or genetic drift could be a disadvantage, but currently, efforts are being made to reproduce the same cell culture conditions to allow the usage of Caco-2 cells as intestinal barrier models among different laboratories (15, 20). Caco-2 cells differentiate into mature enterocytes in 14 to 21 days if cultured on permeable support (20). Researchers have made many attempts to improve the culture protocols and reduce the differentiation times. Chong et al. (23) claimed that using a BioCoat® intestinal epithelial-cell environment, they obtained a Caco-2 monolayer suitable to be an absorption model in only 3 days. Lentz et al. (24) also developed a rapid culture protocol in which iron, different growth factors, and hormones were added to the culture media cells, causing them to differentiate in only 4 days. In 2014, Cai et al. (25) reported that Caco-2 cells cultured on a three-dimensional extracellular matrix substrate using an optimized serum-free medium (containing butyric acid and MITO®) could differentiate in 7 days. These cells displayed comparable cellular morphology and integrity as the traditional 21-day model without significant differences in para-cellular and trans-cellular permeability. However, the human cancer origin of Caco-2 led researchers to isolate and immortalize some intestinal epithelial cell lines from common production animals that can grow on permeable supports and could allow us to better understand the effect of natural bioactive compounds on the animal intestine.

### Cell Lines for Production Animals

Cell lines derived from the intestine of farm animals are rarely available. Moreover, the identity of farm animal cell lines is not so clear. For example, chicken B6 and B10XI cell lines have been identified as porcine cells, and IPEC (an apparent subclone of the IPEC-J2) is now assumed to be derived from cattle (26).

#### Porcine: IPEC-1, IPEC-J2, PoCo83-3 and ZYM-SIEC02

Two non-transformed cell lines, IPEC-1 and IPEC-J2, were established from 12-h-old piglets in 1989 (27, 28). These two cell lines have a different morphology: IPEC-1 has a cobblestone morphology, and IPEC-J2 has an elongated phenotype with a higher cell area (29). In contrast to Caco-2 cells, these cell lines cannot escape attachment-regulated apoptosis (called anoikis), which prevents uncontrolled proliferation (30). Nossol et al. (31) reported a significant upregulation of p53 and other differentiation pathways in IPEC-J2, which can explain the huge proliferative capacity of this not transformed nor tumorigenic cell line. These cell lines can grow and express a TJ network if cultured on permeable support (32). IPEC-J2 is the most commonly used cell line for *in vitro* studies concerning the effect of botanicals on the pig intestine (33–36).

In 2017 Kaiser et al. (37) established a new porcine colonic cell line PoCo83-3. Cells were isolated from the proximal colon of a 3-week-old piglet and transduced using a recombinant retroviral vector construct containing the simian virus 40 large T antigen. PoCo83-3 showed epithelial cell-specific features, and the expression of keratin 18, E-cadherin, and the tight junction-associated proteins. To validate PoCo83-3 as an *in vitro* model in epithelial barrier research, proinflammatory cytokine-inducible alterations in barrier integrity were demonstrated by incubating the cells with TNF-α and IFN-γ for 48 h.

In 2014, Wang et al. established a porcine intestinal epithelial cell line (ZYM-SIEC02) by introducing the human telomerase reverse transcriptase gene into small intestinal epithelial cells derived from a neonatal, 1-day old piglet. ZYM-SIEC02 retained the morphological and functional characteristics typical of primary swine intestinal epithelial cells (38). Recently, these cells have been used to study the protective effect and mechanism of carnosol on pig intestinal oxidative stress (39).

#### Bovine: BIEC and FBCEC

In 2000, Föllmann et al. isolated and established for the first time a bovine intestinal epithelial cell (BIEC) culture from the bovine colon (40). Then other research groups isolated established other BIEC cultures (41–45). Researchers have used BIEC to study some pathogenic challenges such as rotavirus infections (45), toxins (46), short-chain fatty acids, (47), or other pathogens (41, 48–51). In 2019 Katwal et al., immortalized for the first time BIEC line using different methods (52). In particular, Katwal et al. (53) isolated primary BIEC from ileal tissue fragments from a 2-day old dairy calf. Then they purified the cultures from fibroblasts using a limiting dilution method and then immortalized them using SV40 large T-antigen, hTERT, or HPV E6 proteins. These cells have been used to test their susceptibility to enteric pathogens, and TLR mediated immune responses.

Moreover, Kaushik et al. (45) isolated fetal bovine colon epithelial cells (FBCECs) for the first time. They isolated the epithelial cells from two 110–130-day-old fetuses, then the cultured cells were evaluated for susceptibility to enteric viral infection. Immunohistochemical staining for cytokeratin confirmed that 60–75% of cultured cells were epithelial cells. Furthermore, following infection with bovine rotavirus (BRV) over 80% of cells in the ileal and jejunal cultures contained viral protein at 16 h post-infection. Kuroda et al. (54) successfully immortalized these cells in 2015 and found that these cells are also susceptible to *Salmonella* infections.

#### Poultry: Primary Cells

For poultry, where immortalized cells are not available, several research groups have tried to isolate primary intestinal epithelial cells. Immerseel et al. (55) created, for the first time, a primary chicken colonocyte culture able to be infected by *S. enteritidis*, starting from adult chickens. Dimier-Poisson et al. (56) created a chicken intestinal epithelial cells (cIECs) culture able to be infected by *E. tenella* and positive for E-cadherin and cytokeratin on flow cytometry analysis, starting from chicken 18-day-old embryos. Later, Yuan et al. (57) isolated cIECs starting from 14-day-old embryos using the enzyme collagenase type I to recover intestinal aggregates able to generate a monolayer that survived until 9 d in culture; however, cell characterization was missing. Both Kaiser et al. (58) and, more recently, Bai et al. (59), isolated cIECs with a proper morphology, but they degenerated after 7–10 d in culture without reaching confluence. Kaiser et al. also compared monolayers isolated from embryonic and adult intestines, demonstrating no difference in growth. Despite that, the excessive mucus production of adult tissues makes them less suitable than embryonic tissues for cIECs isolation (58). Moreover, Bar Shira and Friedman (60) isolated cIECs starting from 17-day-old embryos and obtained a culture positive for villin and E-cadherin. They showed that these cells could take up and process bacteria and respond to bacterial products (LPS and LTA), and they express proinflammatory cytokine genes (interleukins 6 and 18) and the acute-phase proteins avidin, lysozyme, and the secretory component derived from the polymeric immunoglobulin receptor. Rath et al. (61) proved that it is also possible to create a chicken intestinal epithelial cell line from the intestinal tissues of adult chickens and to maintain these cells through 6-7 passages. However, these cells lack proper epithelial morphology since IF characterization showed that Zonula occludens-1 (ZO1) was not located in their intercellular junctions, a typical trait of intestinal enterocytes. Recently, Ghiselli et al. (62) developed a protocol that required various growth factors to culture cIECs. They showed that it is possible to maintain these cells for up to 14 days in culture. These cells showed proper morphology and TJs localization. They characterized the cIECs for various intestinal markers, such as ZO1, cytokeratin 18, and E-cadherin, and cultured them on a permeable support. Unfortunately, creating a reproducible protocol to create an *in vitro* model with primary cells isolated from fresh intestinal tissue remains a challenge (63). Organoid technology has changed the research landscape, focusing the efforts of research teams on creating models that can closely represent the *in vivo* situation (64).

### Enteroids From Production Animals

Barker et al. (65) identified, for the first time, intestinal stem cells (Lgr5+) in small intestinal and colonic crypts. These cells can differentiate into all intestinal epithelial cells, and they can be cultured *in vitro* for long periods, forming “mini guts” or spherical 3D organoids (66, 67). 3D organoids are useful tools to study epithelial cell differentiation, function, and host-pathogen interactions. Unfortunately, they are heterogeneous in terms of viability, their shape limits bioactive compound penetration, and they are usually unsuitable for food supplement screening and permeability studies (68–70). To overcome this issue, organoid-derived monolayers were developed. Organoid-derived monolayers (or enteroid monolayers) are 2D cultures derived from Lgr5+ stem cells (71), and they were obtained by digesting 3D organoids or seeding isolated intestinal crypts. Enteroid monolayers can be cultured on permeable supports, and they can reproduce all of the different epithelial cells present in the *in vivo* intestinal tissue (72). The idea is to seed dissociated cells obtained from organoids or crypts on Transwell^®^ inserts coated with extracellular matrix proteins (i.e., collagen). Here cells produce a tight 2D monolayer and epithelial differentiation can be induced by removing niche staminal factors or by using an air-liquid interface, as shown for pig enteroids (73). Hee et al. (74) created a porcine enteroid monolayer cultured on Transwell® filters by enzymatically dissociating a 3D organoid. This enteroid monolayer can form a TJ network with high transepithelial electrical resistance (TEER) in 3 days, thus representing a robust platform for exploring intestinal health and permeability and bioactive compound testing in swine (74). In 2019, Töpfer et al. created a bovine colonoid from colonic organoids creating a monolayer with a TEER value of 324 Ω^*^cm^2^ (75). Recently, Resende et al. (76) utilized the same approach using a porcine enteroid monolayer to study the effects of *Lawsonia intracellularis* infection. For other production animals, enteroid monolayers have not yet been developed. Organoids and enteroids can be considered the most accurate tool to study gut health and, in the future, they will be largely used as the golden standard model.

## *In vitro* Co-culture Models

An ideal *in vitro* model must resemble the key characteristics of the intestinal epithelium (13). Epithelial cells seeded on permeable supports can be cocultured with other cell types to mimic a more realistic response to different apical stimuli (77). HT29 is another human colorectal adenocarcinoma cell line that was isolated in 1964 (78). This cell line contains mucus-producing goblet cells. In particular, they can express both secretory (MUC2, MUC5AC, MUC6) and membrane-bound (MUC1, MUC3, MUC4) mucin types (79). HT29-derived cell lines (HT29-MTX) cocultured with Caco-2 cells are a valuable tool to study mucin activity and mucus effects on interactions with botanicals, pathogens, or microflora (15). Arranz et al. (80) tested the absorption across the intestinal epithelium (through Transwell® permeable support) of encapsulated rosemary extract on both Caco-2 and Caco-2/HT29-MTX cocultures. They found that when cocultures were employed, the presence of mucus caused higher retention in the apical layer compared to the Caco-2 monolayer. Volstatova et al. (81) used a Caco-2/HT29-MTX coculture to study the effect of antioxidant compounds (apple, tea, and coffee polyphenols) on mucin expression. The results showed that each polyphenol compound induces different expression patterns of mucin genes. Schimpel et al. (82) found that the best ratio of Caco-2 cells to HT29-MTX cells was 70:30. This ratio granted permeability results near those obtained from *ex vivo* permeability assays using porcine intestinal mucosa. In this study, Schimpel and colleagues replicated a triple-coculture model developed in 2013 by Antunes et al. This model consists of a triple co-culture of Caco-2 cells with Raji B cells and HT2-MTX (83). Raji B is a cell line that originated from a human Burkitt's lymphoma that, if cultured on the basolateral side of a permeable membrane, can induce an M cell phenotype in Caco-2 cells cultured on the apical side (82–84). Another important intestinal barrier function is the immune response. Culturing Caco-2 cells or, more generally, epithelial cells on the apical side of permeable support allows coculture of basolateral side immune cells. This coculture can mimic the immune response to a challenge (85). Cocultivation of Caco-2 cells with monocyte-derived dendritic cells (85), Raji B cells (M-cells) (82), or THP-1 cells (macrophages) (86) allows us to study communication between immune cells and epithelial cells. The ENS is another important player in intestinal physiology. In 2001, Satsu et al. showed that coculturing Caco-2 cells with PC12 cells (a neural cell line derived from a pheochromocytoma) can reproduce the interactions between the enteric nervous system and epithelial cells (87). Finally, it is possible to coculture all of the previous models with probiotics, pathogens, and other bacteria to study the interaction between the intestinal barrier and the microflora (88).

## *ex Vivo* Models

**Figure 2:**
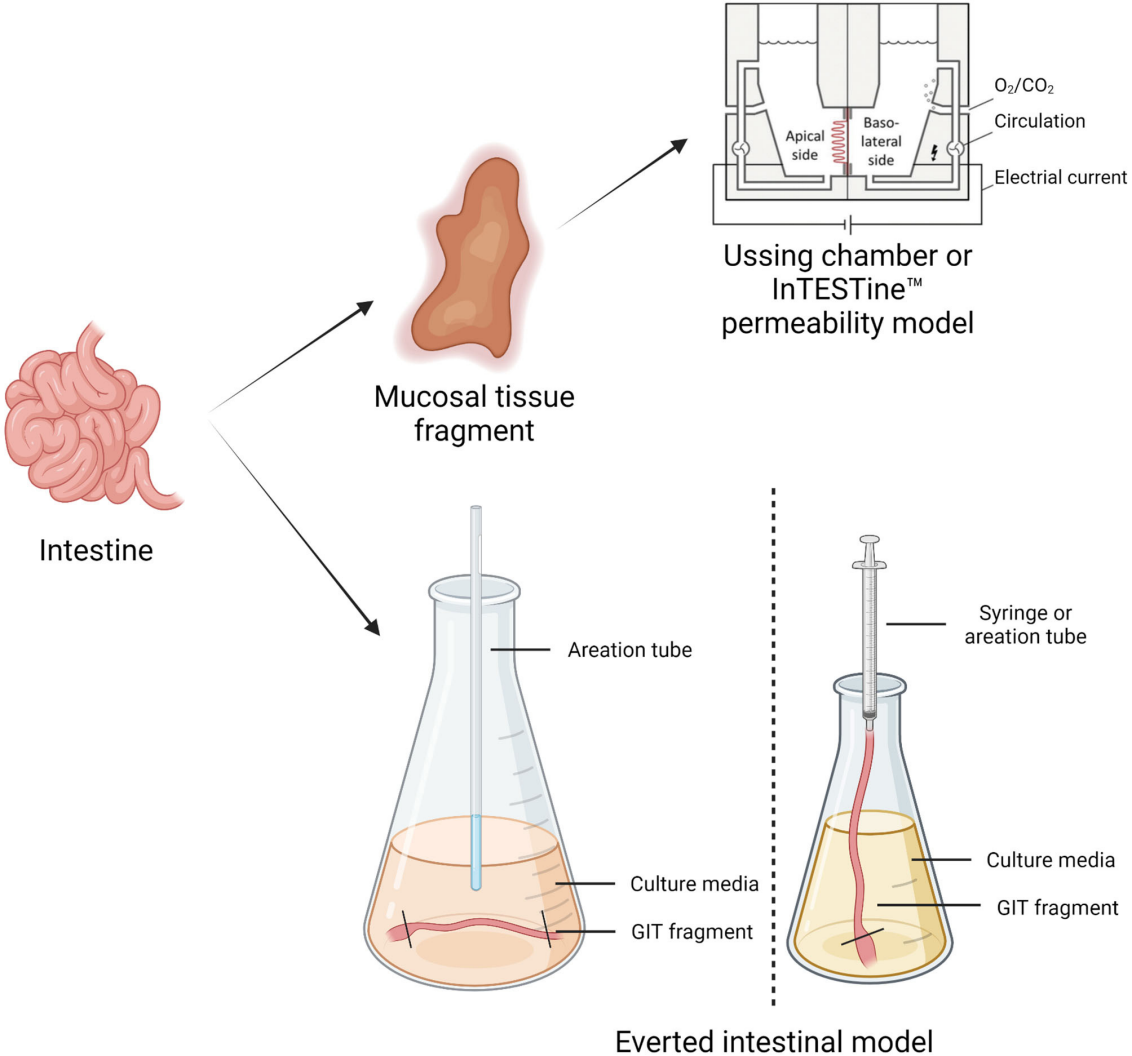
The figure represents the different *ex vivo* models. The Using chamber or the inTESTine™ system works by removing a segment of intestinal mucosa from an animal, which is then mounted between two halves of a chamber filled with physiological buffer to simulate the mucosal and basolateral passage flow. In everted intestinal rings, the animal's intestine is cut into ring slices and transferred into oxygenated culture media. This model is a useful tool for investigating uptake into intestinal cells and metabolism in different regions of the intestine—Created with BioRender.com.

*Ex vivo* models are living functional tissues or organs cultivated in an artificial environment outside the organism (89). Table 1 reports a summary of the benefits and limitations of different *in vitro* and *ex vivo* models. Figure 2 illustrates the different *ex vivo* models.

**Table 1:** *In vitro* and *ex vivo* model benefits and limitations.

**Model**	**Benefits**	**Limitations**
Caco-2 (*in vitro*)	•Can be polarized •Cost-effective •Easy to use •Extensive literature available •Commercially available	•Cancerous origin •Long times to differentiate •Genetic drift
IPEC-J2—IPEC-1 (*in vitro*)	•Can be polarized •Commercially available •Good for studying the small intestine •Non-cancerous origin	•Not a suitable model for the colon
BIEC and FBCEC (*in vitro*)	•Good for studying bovine pathogens •Non-cancerous origin	•Not commercially available •Few papers available •Uncertain polarization capacity
Primary intestinal epithelial cell lines (*in vitro*)	•Can be polarized •Contain multiple cell types •Closer to an *in vivo* situation •Non-cancerous origin Physiologic relevance	•Not commercially available •Expensive maintenance •Short lifetime (weeks) •Needs to sacrifice animals to start a new culture
Organoids/enteroids (*in vitro*)	•Can be grown 2D or 3D •Can be polarized •Contain all epithelial cell types •Closer to an *in vivo* situation •Non-cancerous origin Physiologic relevance	•Not commercially available •Very expensive maintenance •Difficult to obtain and manage •Short lifetime (weeks) •Needs to sacrifice animals to start a new culture
Ussing chamber (*ex-vivo*)	•Tissue is polarized •Can obtain barrier function and transport data •Contain all epithelial cell types •Physiologic relevance	•Short lifetime (<5 h) •Require expensive equipment and knowledge •Needs to sacrifice animals
Everted intestinal ring	•Absence of maintenance •Closest to *in-vivo* situation •Contain all epithelial cell types	•Short lifetime (<3 h) •Needs to sacrifice animals •Muscularis mucosa and lamina propria presence
InTESTine™	•Tissue is polarized •Can obtain barrier function and transport data •Physiologic relevance •Contain all epithelial cell types •Cheaper than Ussing chamber	•Short lifetime •Needs to sacrifice animals

### Ussing Chamber

Ussing and Zerahn (90) developed the Ussing chamber to study transepithelial ion transport across frog skin. Later, Grass and Sweetana (91) adapted it to test the intestinal permeability of drugs. The Ussing chamber works by removing a segment of intestinal mucosa from an animal, opening it to form a flat sheet between two halves of a chamber filled with physiological buffer (95% O_2_, 5% CO_2_, 37°C) (92, 93). Then, by applying a current, it is possible to measure some electrical values that can be indicators of intestinal health (94). Using a fluorescent tracer (such as FD4 or lucifer yellow), it is also possible to measure the para-cellular flux (92). The Ussing chamber is the most commonly used *ex vivo* system for production animals and it has been used for many applications: to test the effect of botanicals and organic acids (95), to evaluate alternatives to in-feed antibiotics (96), to determine the impact of microbes and probiotics on intestinal ion transport (97), and to study transepithelial transport and intestinal permeability (92). Table 2 reports typical TEER values of the different intestinal tracts.

**Table 2:** TEER values for different cell lines, cocultures, and intestinal tracts.

**Cell or Tissue**	TEER (Ω*cm^2^)	**Equipment used**	**Reference**
Small Intestine (*ex-vivo*)	50–100	Ussing chamber	(98)
Colon (*ex-vivo*)	300–400	Ussing chamber	(98)
Caco-2	1,100–1,350	Millicell-ERS system	(99)
Caco-2/HT29-MTX	100–300	Millicell-ERS system	(100)
Caco-2/Raji B	80–100	Millicell-ERS system	(101)
Caco-2/HT29-MTX/Raji B	50–70	Millicell-ERS system	(101)
Caco-2/THP-1	80–100	Millicell-ERS system	(86)
Caco-2/PC12	80–100	Millicell-ERS system	(87)
IPEC-J2	7,000–8,000	Millicell-TERS-electrode	(31)
IPEC-1	6,000–7,000	Millicell-TERS-electrode	(31)
PoCo83–3	4,000–6,000	EVOMX ohmmeter	(37)
Bovine colonoid monolayer	300–400	Chopstick-electrode epithelial voltohmmeter EVOM2	(75)
Porcine enteroid monolayer	1,000–1,150	cellZscope®	(74)
Primary cIECs	50–70	Millicell-ERS system	(62)

### Everted Intestinal Ring

In everted intestinal rings, the animal's intestine is cut into ring slices (30–50 mg, 2–5 mm width) and put into oxygenated media (102, 103). This model is a useful tool for investigating uptake into intestinal cells and metabolism in different regions of the intestine (104). The simplicity of this method, the absence of maintenance, and the closeness to an *in vivo* situation are the principal advantages of this method (103). A disadvantage of this approach is the presence of the muscularis mucosa, which is not usually removed. Therefore, this model does not reflect the actual intestinal barrier, because compounds under investigation pass from the lumen into the lamina propria. Unfortunately, only a few papers written in the 1960s are available regarding the use of everted intestinal rings in production animals (105, 106).

### InTESTine™ Permeability Model

Voortman and colleagues in 2012 described a high-throughput system using *in vitro* intestinal segments from pigs (107). The tissue was incubated in a 24-well culture plate to study the effects of fatty acids on the release of gastrointestinal hormones in pigs (107). This model has been adjusted and commercialized as InTESTine™, which uses porcine intestinal mucosal explants to investigate intestinal absorption (108, 109). This model allows us to test compounds directly on the intestinal epithelium and measure transport and/or secretion across the epithelial tissue. Moreover, this setup can be used in a humidified oxygenated incubator at 37°C on a rocker platform (109). This system has been used to study intestinal physiology (110), chemosensing (111), microbiota interactions (108), and intestinal stem cells (112).

## *In vitro* and *Ex vivo* Assays

Intestinal permeability assays are commonly used as indicators of “gut barrier health” (113). Paracellular permeability and transepithelial electrical resistance, molecular approaches (i.e., qPCR or ELISA), and immunofluorescence assays (IF) are common ways to assess the intestinal barrier status *in vitro* and *ex vivo*.

### Tracers and Paracellular Permeability

The paracellular permeability assay (PCP) consists of quantifying the passage of a tracer molecule through the paracellular route, typically used by medium-sized hydrophilic molecules (≤ 600 Da *in vivo*; ≤ 10 kDa *in vitro* in cell lines) from the luminal side to the basolateral side of the intestinal epithelium (114). PCP can be performed both *in vitro* (culturing cells on a permeable system) or *ex vivo*. In the 1990s, a paracellular permeability assay on human jejunal segments was performed with the Ussing chamber for the first time (115). Hubatsch et al. (116) explained how to perform a permeability assay on Caco-2 cells cultured on filters. This protocol can be shifted and readapted for every intestinal epithelial cell line that can grow on permeable supports. During PCP, tracers are monitored using a fluorescent dye (e.g., fluorescein isothiocyanate) bonded typically to a sugar (e.g., mannitol or dextran) that can pass through the epithelium only using the paracellular method (16). Tracer molecules are also commonly used *in vivo*. They are non-digestible sugars such as labeled dextran (FITC-d), lactulose, 51Cr-EDTA, mannitol, or PEG (117) which can be later quantified in urine or blood. These methods are widely used for example in poultry [recently well described by Liu et al. (118)] but the output in most of these techniques is a single value of permeability, which does not allow to distinguish which region of the gut is affected, and therefore must be used in combination with other methods (117). These molecules can be used in *ex vivo* explants (i.e., Ussing chamber) allowing evaluation of very specific regions of the GI tract. However, tissue viability is a big concern, and therefore incubation times no longer than 3 h are recommended (119). Dextran is a non-digestible sugar (>1 kDa), and it represents a typical agent that is transported *via* the paracellular route (120, 121). Transcytosis of dextran has never been reported. However, dextran pinocytosis was observed, but it is a very slow process and could be ignored compared to its paracellular transport (122). The PCP is measured as a ratio between the fluorescence read on the apical side and the fluorescence read on the basolateral side (123). Before starting an experiment, a standard curve was generated to relate the tracer molecule concentration to the fluorescence emission (124). A healthy epithelium will retain most of the tracer molecule in the apical part, while a damaged epithelium will leave the tracer free to move to the basolateral part. For example, during a challenge, it is important to perform PCP using tracers with different molecular weights to reveal the size selectivity of TJs and to see if this has been compromised (125). Van Itallie et al. (126) reported that modest permeability changes are more difficult to detect with large tracers (>10 kDa) except in cases where the barrier is catastrophically damaged. Using smaller molecules (<4 kDa), it is possible to detect less extreme barrier damage (127). PCP using dextran is used to determine the effect of botanicals (34, 95), oxidative stress (128), or inflammatory (129) challenges on the TJs network and, more generally, on animal intestinal health (130).

### Transepithelial Electrical Resistance and Short Circuit Current

Transepithelial electrical resistance is a rapid, non-invasive method for quantifying barrier tissue integrity by measuring electrical resistance across the epithelium. TEER can be measured in both *in vitro* and *ex vivo* models. There are two principal ways to measure TEER: resistance-based measurements and impedance-based measurements (131). *In vitro*, resistance-based measurements are performed by placing “stick electrodes” on the apical and basolateral sides of a cell layer grown on a semipermeable membrane and by applying an alternate current signal (12.5 Hz and 10 μA). *Ex vivo* (i.e., an Ussing chamber), the potential difference across the epithelial tissue can be determined using Ag/AgCl electrodes. Then, the TEER value is determined using Ohm's law: measuring both current and voltage across the cell layer or the potential difference across the epithelial tissue. Resistance is given by the combination of the transcellular and paracellular pathways. Different factors, such as medium resistance, electrode-medium interfacial resistance, and the resistance of the semipermeable membrane (in *in vitro* assays), contribute to the TEER. A simple approach to reduce these variables is to subtract from all resistance measurements the resistance of an identical testing configuration without the cell layer (a blank) (131). Impedance-based measurement is a more advanced technique that can allow for the determination of TEER in a more robust manner (131). Measurements are taken across a range of current frequencies, and the resulting impedance (resistance in an alternate current circuit) is plotted as a function of frequency. The resulting total impedance value provides information not only about the TEER but also about the capacitance (ratio of the change in electric charge over the corresponding change in electric potential) of the cell layer or tissue, which can be a readout parameter (132). The software can automatically determine the best-fit parameters and extract the TEER using standardized cell support models (132). Table 2 reports the typical TEER values of the cell lines and intestinal tracts.

Another important parameter is the short circuit current (ISC). ISC is measured in *ex vivo* assays using the Ussing chamber (92). When performing the assay, it is possible to use, in one of the two halves, a buffer without specific ions (i.e., Na^+^ or Ca^2+^). Naturally, the epithelium, using ion pumps, balances the ion concentration between the two sides, generating a potential difference. Applying a certain external current, the ISC, it is possible to nullify this potential difference, creating a “short circuit” (92). ISC, as well as TEER, is influenced by the epithelial barrier status: a healthy epithelium will have a higher ISC than a disrupted epithelium (90).

### Immunofluorescence Assays

To visualize the health status of the epithelium *in vitro*, it is also possible to use immunofluorescence (IF) or molecular approaches. IF allows us to take a picture of the health condition of the examined epithelium during a challenge or treatment. Kawauchiyaa et al. (133), in their research article, showed a correlation between the decrease in TEER values and ZO1 degradation by IF. Treating a Caco-2 cell culture with patulin, an antibiotic molecule, caused a significant decrease in TEER values and a complete disappearance of ZO1 in the IF assay (133). Van Itallie et al. (126) demonstrated the same results in MDCK cells. The absence of ZO1 expression in knockout cells (as observed by IF) was correlated with a higher PCP and a loss of normal epithelial cell morphology. Moreover, other molecular assays, such as qPCR or ELISA, are available and cost-effective alternatives to investigate the effect of botanicals on protein or cytokine expression (134).

### Different TJs, Different Assay

PCP and TEER are two of the most commonly used assays to investigate the barrier function of an intestinal model *in vitro* or *ex vivo*. Different TJs proteins influence TEER and PCP assays (135). Claudin-based pores (136, 137) and occludin (138) influence the TEER, which measures the ion flux through the epithelium. Alternatively, PCP measures the flux of larger molecules *via* a route called the “leaky pathway” (1, 126, 139), which is principally affected by ZO1 expression. Suzuki et al. (138) treated Caco-2 cells with kaempferol, a flavonoid found in kale, beans, tea, spinach, and broccoli (140). This treatment increased claudin-3 and occludin expression in IF. Consequently, the TEER values increased, but PCP was not affected (138). Endo et al. (141) also confirmed the correlation between decreased claudin expression and decreased TEER values. Van Itallie et al. (126) demonstrated that the absence of ZO1 expression observed by IF (in MDCK knockout cells) was correlated with a higher PCP and a loss of normal epithelial cell morphology, but the TEER values were not affected. Last, Mani et al. (142) reported a correlation between increased TEER and increased expression of claudin-3 and claudin-4 after treating heat-stressed IPEC-J2 cells with zinc butyrate.

## Discussion

In this review, a descriptive analysis of *in vitro* and *ex vivo* models available to study animal intestinal permeability and health was performed.

First of all, an essential discussion point concerning *in vitro* models is the origin of the cell culture. Caco-2 cells are the “gold standard” cellular model for *in vitro* intestinal barrier assays, even for animals (16). But these cells are derived from a human colorectal carcinoma (17). These cells are cheap, easy to use, and maintain. Caco-2 cells are easy to culture and they can differentiate into mature enterocytes spontaneously when reaching confluence. The origin of these cells is a crucial limitation because some pathogens and some pathways or microbial interaction could be not so close to reality due to their origin. This limitation is also applicable to other human cell lines used in animal studies i.e., HT29 used in co-culture models. In this context, some continuous cell lines derived from pigs and bovines have been established. Their non-cancerous origin is the major benefit of these cell lines, and they better represent a pig or bovine small-intestinal epithelium. Moreover, immortalized cell lines are considered the most cost-effective tool because they can be passaged indefinitely, but due to the different culture conditions and different numbers of passages among different laboratories, immortalized cells could often acquire different properties overtime. Thus, the expression of different markers on the enterocytes, can change with increasing numbers of passages. Also, parameters like TEER and proliferation rate have been reported to increase with passage number (143). Primary cells are often considered to be more biologically and physiologically similar to *in vivo* situations. But they are more expensive to maintain, and they have a limited lifespan, requiring frequent isolation from live tissue.

Another interesting discussion point is the epithelial composition. The normal intestinal epithelium is made up of several cell types and differences in gene expression profiles are not only observed in the mucosal epithelium along the GIT but also along the crypt-villus axis. Experiments done with monotype-monolayers cannot be directly compared with the *in vivo* situation. However, they can be an ideal way to study molecular mechanisms in a simplified environment. Lastly, the absence of a mucus layer above the cells could be another limitation in the study of enterocytes/microbial interactions that can affect intestinal permeability and health. To overcome these problems many efforts have been made to create enteroid monolayers, 3D organoids, and more complex systems for production animals (144), despite the high cost and particular medium or support necessary to culture them. The 2D enteroid and 3D organoids could be a valid alternative to have a closer cellular composition to the *in vivo* situation. Moreover, specific differentiation methods have allowed researchers to examine cell-type-specific responses and properties including barrier function, and would be useful tools to examine host-microbe interactions.

As previously described, it is better to use primary cells or organoids, preferably derived from the animal of interest, as they carry specific properties of the animal species they derive from. *Ex vivo* systems can add complexity and functional crosstalk between cell types that are not present in *in vitro* systems (143). The InTESTine™ system contains a mucus layer that enables it to be more successfully utilized in conjunction with single or mixed communities of bacteria. Unfortunately, thus model been validated for this specific purpose yet.

The Ussing chamber is widely used for these assays. It uses live intestinal mucosal tissue that can be treated before or during the assay, and multiple parameters can be measured. The Ussing chamber can be also a valid system to study the intestine/microbiota interaction. For example, it has been used to study bacterial translocation into colonic mucosa (145). In another study, *Clostridium difficile* was tested in a Ussing chamber to study the interactions with host epithelial cells and the bacterial and toxin-mediated cellular events (146). However, the short tissue viability is the major disadvantage of this system, which cannot be used for longer-term studies (>5 h). Nevertheless, the *ex vivo* models do not permit a more sequential and basic approach provided by the other *in vitro* assays. Thus, they must be seen as a complement and not as an alternative to *in vitro* studies. Furthermore, they can be more difficult to implement.

In comparison to intestinal epithelial cell lines, intestinal organoids and 2D enteroids contain all the different specialized cell types of the intestinal epithelium. Thus, intestinal organoids represent a promising tool for studies on specific regions of the intestine. Seeger in 2020 widely discussed the usage of organoids derived from farm animals (147). For certain studies (i.e., bioactive molecule testing) the use of standardizable monolayers is more appropriate than the use of organoids. In humans, it is also possible to generate intestinal organoids and derived monolayers from human pluripotent stem cells (148). Actually, no published studies exist on the generation of intestinal organoids differentiated from pluripotent stem cells of farm animal species. In 2021 Kumar et al. (149) well discussed the current achievements in the derivation of pluripotent stem cells from farm animals, and discuss the potential application areas. Another interesting perspective is the usage of 3D scaffolds. A first attempt was made by Sala et al. (144). Jejunal crypts of pigs were engrafted intraperitoneally on biodegradable scaffold tubes and examined for the cell types present after seven weeks (144). The resulting organoids had a columnar epithelium expressing enterocytes, goblet cells, and intestinal stem cells. They were surrounded by intestinal subepithelial myofibroblasts, representing the lamina propria and smooth muscle cells with some neuronal cells among them, representing a lamina muscularis layer. These advanced *in vitro* models are not as complex as the *in vivo* situation, but they allow the investigation of interactions between the surrounding cell types and the intestinal epithelium in a more complete manner.

## Conclusion

All of the models and techniques illustrated in this review could be useful for investigating and better understanding the interactions among the different intestinal components. Moreover, *in vitro* or *ex vivo* models are needed to elucidate the mechanistic foundations and physiological significance of beneficial or pathogenic relationships between the GIT epithelial barrier and the external environment (9), creating well-controlled and repeatable conditions. A healthy intestinal barrier allows the maintenance of mucosal immune homeostasis and prevents the onset of uncontrolled inflammation. The usage of *in vitro* or *ex vivo* models could be an optimal way to study strategies to improve the gut barrier in compliance with the 3Rs approach.

## Author Contributions

FG, BR, AP, and EG equally contributed to the conception and creation of the review. All authors read and approved the final manuscript.

## Conflict of Interest

BR was employed by company Vetagro S.p.A. AP and EG serves in the board of Directors of Vetagro S.p.A. The remaining authors declare that the research was conducted in the absence of any commercial or financial relationships that could be construed as a potential conflict of interest.

## Publisher's Note

All claims expressed in this article are solely those of the authors and do not necessarily represent those of their affiliated organizations, or those of the publisher, the editors and the reviewers. Any product that may be evaluated in this article, or claim that may be made by its manufacturer, is not guaranteed or endorsed by the publisher.

## Abbreviations

BIEC: Bovine intestinal epithelial cell
cIECs: Chicken intestinal epithelial cells
ENS: Enteric nervous system
FBCECs: Fetal bovine colon epithelial cells
GIT: Gastrointestinal tract
IF: Immunofluorescence
ISC: Short circuit current
PoCo83–3: Porcine colonic epithelial cells
PCP: Paracellular permeability
TEER: Transepithelial electrical resistance
TJs: Tight junctions
ZO1: Zonula occludens 1.
